# Ultrasound-Guided Morcellation During Holmium Laser Enucleation of the Prostate

**DOI:** 10.1089/cren.2018.0057

**Published:** 2018-08-01

**Authors:** David T. Tzou, Ian S. Metzler, Marshall L. Stoller, Thomas Chi

**Affiliations:** Department of Urology, University of California, San Francisco, San Francisco, California.

**Keywords:** HoLEP, ultrasound, morcellation, BPH

## Abstract

***Background:*** Holmium laser enucleation of the prostate (HoLEP) has emerged as an accepted standard of care for the treatment of benign prostatic hyperplasia. This surgery relies on morcellation of the prostate adenoma once enucleation of the transition zone has been completed. Caution is required during this portion of the operation, as engaging bladder mucosa within the morcellator can result in bladder injury, a rare but potentially catastrophic complication of HoLEP. Morcellation of the prostatic tissue can be additionally challenging if visualization is poor from either equipment failure or increased bleeding from a highly vascularized prostate.

***Case Presentation:*** We report the case of a 66-year-old Caucasian man with an estimated 158 g prostate who underwent HoLEP at our institution. Enucleation was uneventful; however, upon placement of the nephroscope to begin morcellation, it was immediately evident that the lens of the nephroscope was damaged as there was extremely poor visualization. Without a replacement nephroscope available, this would have normally resulted in aborting the case and returning another day to complete the morcellation. Concurrent bladder ultrasonography was performed and allowed for additional visual feedback to the operator, helping guide the morcellator to safely engage the enucleated adenoma and complete the operation.

***Conclusion:*** This case report demonstrates the ability of performing the morcellation portion of HoLEP mainly with the visualization provided by concurrent bladder ultrasonography. By providing additional imaging feedback to the operator, ultrasound can be a complementary tool to assist in safely performing morcellation in situations of suboptimal cystoscopic visualization during HoLEP.

## Introduction and Background

Holmium laser enucleation of the prostate (HoLEP) is an established standard treatment for men with benign prostatic hyperplasia (BPH). The steep learning curve for this operation has been well documented, attributed to the technical challenges of enucleating the entire transition zone, time needed to complete the operation, and incomplete morcellation.^1^ Although HoLEP has generally been associated with infrequently reported complications,^2^ one serious complication to avoid during morcellation is inadvertently engaging bladder mucosa within the blades that can lead to significant bladder injury. Although direct observation with the nephroscope and dual inflow irrigation to maintain a distended bladder help minimize the likelihood of this injury occurring, it has still been reported to occur in up to 5.7% of reported case series.^3^ We report a case at our institution in which the nephroscope lens was found to be damaged, producing a poor, extremely limited image. Without a replacement nephroscope available, this scenario may have resulted in aborting the operation and returning another day to complete the morcellation. Faced with this challenge, a novel application of concurrent bladder ultrasonography was utilized that allowed for safe completion of morcellation and HoLEP in a single setting. Herein we present this case.

## Case Presentation

The patient is a 66-year-old Caucasian man with a history of an enlarged prostate and urinary retention with a prostate volume of 158 cc on pelvic ultrasonography. After informed consent was obtained, HoLEP was performed using a 550-μm end-firing laser fiber and a 100-W holmium laser (Lumenis, Inc., Tel Aviv, Israel). Laser settings were 2 J and 20 to 40 Hz, translating into 40 to 80 W of power. Enucleation was performed by making a transverse laser incision just proximal to the verumontanum to identify the appropriate prostatic capsule plane. With minimal median lobe tissue present, a 6 o'clock incision was made connecting the bladder neck with the initial transverse incision. The enlarged left and right lateral lobes were then enucleated in the standard manner as described previously.^4^ Transurethral morcellation was initiated using the 26F outer sheath with the Wolf Piranha Scope (12° nephroscope) and the Piranha morcellator (Richard Wolf, Knittlingen, Germany) at the manufacturer's recommended settings of 1500 rpm.

**FIG. 1.**
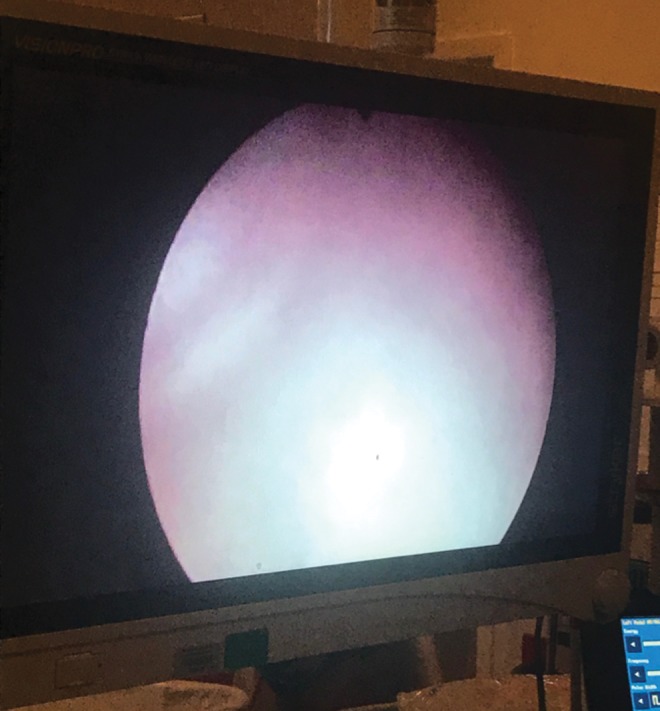
Poor cystoscopic image seen with damaged nephroscope during morcellation.

**FIG. 2.**
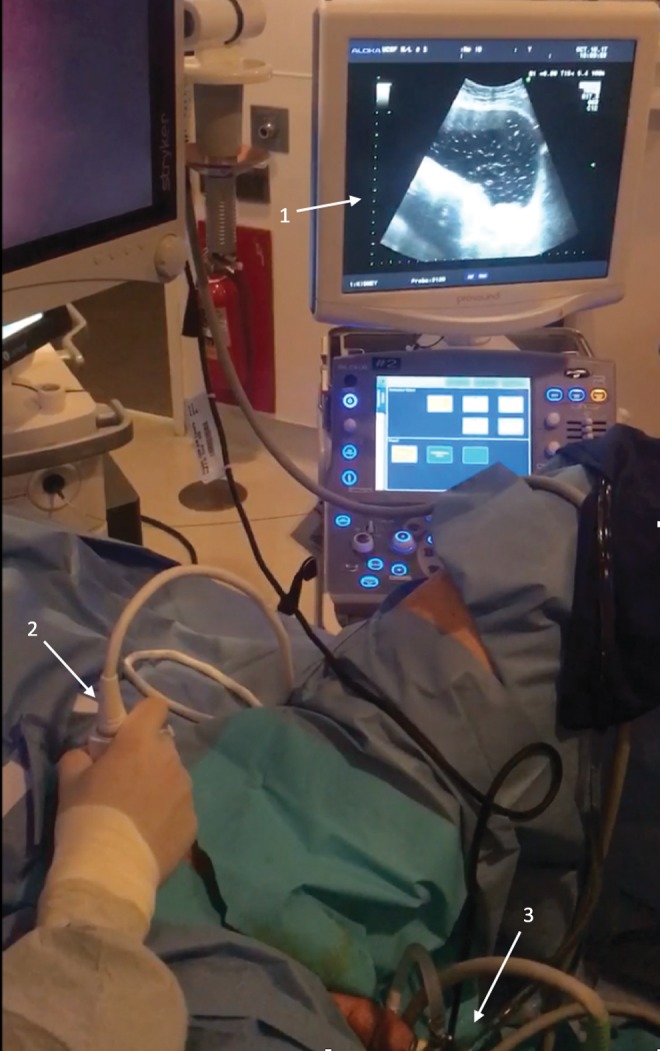
Arrangement of ultrasound machine with use of convex abdominal transducer probe under B-mode ultrasound to observe bladder in sagittal view. 1 = ultrasound monitor and console; 2 = ultrasound probe; 3 = resectoscope.

Upon placement of the nephroscope into the bladder, it became apparent that the lens was damaged resulting in an extremely poor cystoscopic image (Fig. 1). No replacement nephroscope was readily available. To safely complete the procedure, it was decided to utilize a 3.5-MHz convex abdominal transducer (Hitachi Prosound Alpha 7; Hitachi Aloka Medical America, Wallingford, CT) under B-mode ultrasound to view the bladder and guide morcellation (Fig. 2).

**FIG. 3.**
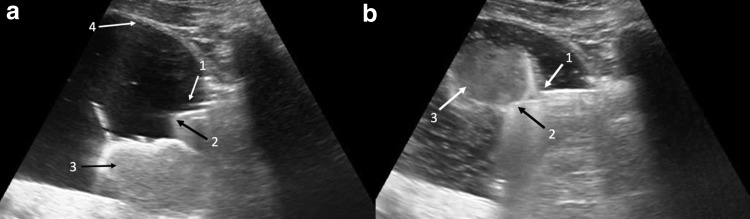
Ultrasonographic images of bladder, sagittal view. *Black* and *white arrows* with corresponding numbers used to label key structures. **(a)** The morcellator can be seen as it is introduced into the bladder. 1 = tip of nephroscope; 2 = tip of morcellator; 3 = dependent enucleated adenoma; 4 = bladder wall. **(b)** The morcellator is now imaged, positioned safely in the middle of the bladder, with the engaged adenoma being morcellated. 1 = tip of nephroscope; 2 = tip of morcellator; 3 = enucleated prostate adenoma engaged in morcellator.

In the sagittal plane, the enucleated adenoma could be seen in the dependent portion of the distended bladder with both the nephroscope and Piranha morcellator just entering the bladder neck (Fig. 3a). Guided by the assistant holding the ultrasound probe and controlling the view, the operator aimed the morcellator tip in the direction of the dependently resting enucleated adenoma, and the suction pedal was applied until the tissue was seen to be engaged into the morcellator blade. The nephroscope and morcellator hand piece were then tilted anteriorly toward the center of the bladder and the morcellation pedal was activated, safely removing the adenoma away from the walls of the bladder (Fig. 3b). A final inspection with both ultrasound and a functional cystoscope lens confirmed the absence of any remaining adenoma pieces within the prostatic fossa and bladder. The patient was safely discharged from the hospital the next day and experienced an uneventful postoperative recovery. Pathology analysis demonstrated a specimen weight of 69 g of BPH. A defective water seal was identified as the source of the defective lens.

## Discussion

To our knowledge, this is the first published report of relying mainly on ultrasound to guide the morcellation portion of HoLEP. In this particular case, visualization was extremely limited because of equipment failure, with the nephroscope lens being severely compromised. As no replacement nephroscope was available, normally this scenario would have resulted in deferring morcellation to another day, thus a return trip to the operating room and another anesthesia event for the patient. Alternatively, an open incision/cystotomy could have been performed to remove the prostate adenoma; however, this would have been more invasive and likely resulted in greater morbidity. This case demonstrates that under ultrasound guidance, morcellation is possible and allows for safe completion of the HoLEP even in the absence of direct cystoscopic observation.

Previous studies have shown that the learning curve for HoLEP can be challenging with commonly cited reasons being the technical difficulty associated with enucleation, as well as the increased time required to perform the surgery.^1^ Although the morcellation portion of the operation can be a relatively safe and straightforward process, it depends on good visualization of the adenoma and the operator being vigilant in suctioning only the adenoma and not the bladder into the morcellator blades. Reports in the literature mentioning superficial bladder mucosal injuries have associated large prostates with increased bleeding, resulting in decreased visualization during morcellation.^2,3^

Given that cystoscopic visualization is generally sufficient to complete morcellation, relying mainly on ultrasound guidance is certainly not recommended for all HoLEP cases. However, ultrasound should be considered whenever there is decreased cystoscopic visualization at the time of morcellation. Especially for surgeons early in their learning curve, ultrasound imaging allows the operator to clearly establish the two essential parameters for safe morcellation: (1) that the bladder is adequately distended and (2) the morcellator tip is positioned in the center of the bladder lumen, away from the mucosa. Ultrasound provides additional visual feedback to the operator with respect to nephroscope location within the bladder and relative to the remaining adenoma, thereby providing an additional safety measure against inadvertently engaging the bladder mucosa.

Meanwhile, as ultrasound machines are readily available in most operating rooms, minimal additional cost is associated with applying this imaging modality during morcellation when needed. The main limitation in applying concurrent bladder ultrasound is that it requires an additional assistant at the bedside. Moreover, to help guide the morcellator operator to the appropriate location, the ultrasound operator needs to be familiar with imaging the bladder and identifying both the morcellator tip and the enucleated adenoma. However, given the clarity of these structures and the ease through which they are usually identified on ultrasound, these skills are readily attainable and useful when the need arises.

## Conclusion

This patient's case illustrates the potential benefit of using concurrent bladder ultrasound at the time of morcellation during HoLEP. Ultrasound provides additional visual feedback to the operator that allows for safe completion of morcellation in situations of suboptimal cystoscopic visualization.

## Abbreviations Used

BPH: benign prostatic hyperplasia
HoLEP: holmium laser enucleation of the prostate
